# Impacts of Maternal Bovine Appeasing Substance Administered at Weaning on Behavioral and Physiological Adaptation of Beef Heifers to the Feedlot

**DOI:** 10.3390/ani15192788

**Published:** 2025-09-24

**Authors:** Désirée Gellatly, Yaogeng Lei, Alison Neale, Lyndsey Smith, Emilie Edgar, Brittany Bloomfield, Brianna Elliot, Irene Wenger, Sean Thompson

**Affiliations:** Technology Access Centre for Livestock Production, Olds College of Agriculture & Technology, 4500 50 Street, Olds, AB T4H 1R6, Canada; dgellatly@oldscollege.ca (D.G.); ylei@oldscollege.ca (Y.L.); aneale@oldscollege.ca (A.N.); smith.lyndsey07@gmail.com (L.S.); eedgar@oldscollege.ca (E.E.); bbloomfield@oldscollege.ca (B.B.); belliot@oldscollege.ca (B.E.); iwenger@oldscollege.ca (I.W.)

**Keywords:** cattle, confinement, coping, stress, weaning, welfare

## Abstract

**Simple Summary:**

Weaning, the separation of cow–calf pairs and cessation of milk intake, is widely regarded as one of the most stressful events in a calf’s life, typically occurring in North America between 6 and 8 months of age. To adapt, calves undergo a range of subtle behavioral and physiological changes, including shifts in feeding behavior and immune activation, aimed at facilitating their adjustment to new social and nutritional environments. This study investigated whether a synthetic analog of maternal bovine appeasing substance (mBAS) administration could help mitigate stress, facilitate feedlot adaptation in beef heifers, and improve production efficiency. Twenty-two Angus-influenced heifers were assigned to receive either 10 mL of mBAS or water (control) at weaning, immediately prior to transportation. Heifers treated with mBAS at weaning exhibited different coping strategies to the feedlot than untreated heifers. They ate more efficiently, spent more time ruminating, gained more weight, and converted feed into growth more effectively than untreated heifers. They also exhibited signs of enhanced immune responses based on blood parameters and appeared to be potentially more profitable than untreated heifers. Administration of mBAS at weaning improved feedlot adaptation in beef heifers, resulting in greater growth and feed efficiency, which may translate into potential profitability.

**Abstract:**

The effects of administering 10 mL of maternal bovine appeasing substance (mBAS) or water (control; CT) at weaning (day 0) before transport on feedlot adaptation and efficiency were evaluated in twenty-two Angus-influenced heifers (*n* = 11/treatment) over 28 days. Body weight (BW), salivary cortisol, blood for complete blood cell count, rectal temperature, chute score and exit speed were collected on days 0, 14 and 27. Intake, feeding duration, frequency and rate, as well as activity and rumination were monitored daily using automated systems. Average daily gain (ADG) and gain-to-feed ratio (G:F) were calculated for each 14-day interval as well as for the entire feeding period. Treated heifers spent less time eating (*p* ≤ 0.06) on weeks 1 and 2, with greater feeding rate and activity (*p* < 0.01) in week 1, followed by reduced activity (*p* ≤ 0.05) in weeks 2, 3 and 4. Rumination was longer (*p* < 0.05) in weeks 3 and 4, coinciding with greater (*p* ≤ 0.05) final BW, ADG_0–27_, ADG_14–27_, and G:F_0–27_, G:F_14–27_. Lymphocyte and hematocrit were lower (*p* < 0.05) on days 14 and 27, respectively, and platelets tended to be greater (*p* = 0.08) than CT for the entire period. Treated heifers achieved numerically greater profit margins than CT. Overall, mBAS enhanced feedlot adaptability post-weaning, improving production efficiency, which may translate into potential profitability; however, this interpretation should be viewed cautiously considering some design limitations.

## 1. Introduction

In the North American beef industry, weaning calves from their mothers, typically between 6 and 8 months of age [1], marks a profoundly stressful transition due to the loss of both maternal presence and milk [2,3]. This initial stress is often intensified by a cascade of other challenges calves encounter around weaning or upon feedlot entry. These can include processing procedures such as ear tagging, vaccination, and growth implants, as well as stress from transportation and auction markets [1]. Over their first days in the feedlot, calves must also adapt to entirely new feed sources and establish new social structures, leading to competition [4]. The cumulative effect of these stressors significantly compromises their immune systems, leaving them highly vulnerable to Bovine Respiratory Disease (BRD), which is the primary driver of morbidity, mortality, and substantial economic losses within the industry [5,6]. To cope with these stressors, calves employ subtle behavioral and physiological adaptations, including altered feeding patterns [2] and immune system activation [3,7], aiming to facilitate their adjustment to the new environment.

Management strategies such as preconditioning, which involves practices like vaccinating calves at least 30–60 days before weaning [8], as well as castrating and dehorning at least three weeks prior to transport, and acclimating them to feed bunks, are known to reduce stress and improve calf health and welfare [1,9]. However, the implementation of preconditioning programs across North America has been inconsistent, largely due to concerns about their cost-effectiveness for both cow–calf and feedlot producers [1,8,10,11]. In Canada, preconditioned calves typically do not receive price premiums, further limiting uptake [1]. As a result, there is a critical need for effective, low-cost strategies that can mitigate stress, support adaptation to the feedlot environment, and enhance the overall welfare of newly weaned calves.

All mammals, including bovine species, possess an evolutionarily conserved mechanism in which maternal appeasing substances (mBAS), a complex blend of volatile and non-volatile compounds such as fatty acids [12,13], are released by specialized sebaceous glands, primarily around the mammary and inguinal regions [14]. These pheromone-like substances play a vital role in establishing cow–calf bonding and promoting behavioral and physiological stability in offspring during stressful events [13]. Multiple studies conducted in the U.S. have demonstrated promising results with synthetic mBAS in beef cattle, a compound that replicates the natural pheromone. When administered at weaning or feedlot entry, improvement on growth performance [15,16,17,18,19] and immune responses [18,19,20] were observed while enhancing feed efficiency [17,19], increasing feed intake [17,18] and reducing physiological stress responses [15,17,18,19,20]. However, the effects on feed intake and feed efficiency remain inconclusive in the literature [17,18,19,20], possibly due to the fact that those previous studies have primarily relied on pen-level average feed intake and/or growth rates, which may mask individual variations. Thus, further studies are needed to evaluate the impact of mBAS on individual-level feed intake, growth, and feed efficiency, along with physiological and behavioral metrics of stress and health that may influence these outcomes in a feedlot setting.

The objective of this study was to evaluate the effects of a synthetic mBAS administration, that has been demonstrated to be effective for up to 15 days [20], at weaning on the behavioral and physiological adaptation of beef heifers during the 28-day feedlot receiving period following transportation, as well as its impact on return-on-investment (ROI). We hypothesized that heifers treated with mBAS would exhibit reduced stress responses, improved feedlot adaptation, and enhanced performance outcomes compared to control heifers, ultimately resulting in a greater ROI.

## 2. Materials and Methods

### 2.1. Animals, Treatment, and Management

All animals were cared for in accordance with policies and guidelines of the Canadian Council on Animal Care [21] after review and approval by the Olds College Institution-al Animal Care and Use Committee (Protocol AUP 096). A total of 22 Angus-influenced heifers (238.1 ± 10.96 days of age), born and housed as a single herd with their dams on pasture at Olds College Smart Farm (OCSF) research facilities (Olds, AB, Canada), were assessed over a 28-day experimental period. On day 0, heifers underwent abrupt weaning, were individually restrained in a hydraulic squeeze chute, weighed (weaning weight, kg) and assessed for reactivity at handling (see details below). Immediately thereafter, heifers were randomly assigned in equal numbers to one of two treatment groups, ranked by age, reactivity at handling, and weaning weight. One treatment group received a maternal bovine appeasing substance (mBAS; FerAppease, FERA Diagnostics and Biologicals, College Station, TX, USA), administered topically to the nuchal area and muzzle (5 mL per site; *n* = 11). The control group (CT; *n* = 11) received a placebo treatment of water administered at the same anatomical site location as the treatment (5 mL per site). The mBAS used in this study consists of a blend of palmitic, oleic, and linoleic acids [20]. To prevent cross-contamination between treatment groups, all handling procedures described below were first carried out on the CT group while the mBAS group remained in a separate holding pen.

Later on (day 0), heifers were handled again for a second weaning weight measurement, baseline sampling (rectal temperature, saliva, and blood; see details below), a second reactivity at handling assessment and treatment administration, and were then held in a pen until all animals in the group had been processed. Immediately following processing, the CT group was loaded into a livestock trailer and transported for 84 km (1 h duration) to simulate the stress of typical hauling conditions experienced by weaned calves in Western Canada [22]. After the CT group had been loaded, the mBAS group underwent the same procedures (day 0), including weighing, rectal temperature, saliva and blood sampling, and transportation in an identical separate trailer. Both groups were subjected to the same transportation conditions (the same space allowance per animal per trailer, the same route, same duration of diet and water deprivations, and a controlled, predesignated speed), until they returned to the same facilities at the OCSF. An automatic recording device was installed inside each trailer’s animal compartment to monitor temperature and humidity. The interval of hauling between groups was limited to approximately 65 min. Upon return, each treatment group was housed in a separate experimental feedlot pen located at a distance of 148 m from each other, and feed was withheld for two hours (resting period).

After the resting period, animals were again herded to the cattle handling facilities at OCSF, where each heifer was individually weighed (shrunk BW, kg), underwent a second-round of rectal temperature and saliva sampling. Immediately after sampling, a radio frequency identification (RFID) ear tag fitted with an automated data logger was applied to the left ear of all animals (see details below). All animals were then vaccinated against Bovine Rhinotracheitis, Viral Diarrhea, Parainfluenza-3, Respiratory Syncytial Virus, and *Mannheimia haemolytica* (Pyramid^®^5+; Boehringer Ingelheim Animal Health USA Inc., St. Joseph, MO, USA), as well as *Clostridium* spp. (Fermicon 7/Somnugen; Boehringer Ingelheim Animal Health USA Inc., St. Joseph, MO, USA). A third reactivity at handling assessment was conducted during this handling event. Following the handling procedures, CT heifers returned to their respective experimental feedlot pen. The same resting period, sampling, and procedures were applied to the mBAS group upon their return at the OCSF (day 0). Additionally, the exact times of treatment administration, loading, unloading, and sampling were recorded. This ensured that both treatment groups were subjected to the same conditions, including an approximately three-hour fasting period encompassing processing, transportation, and resting period.

While housed in the experimental feedlot pens (from day 0 to day 27), animals from both treatment groups were housed in two identical pen environments based on space allocation (~80 m^2^ per animal/pen), and the pen’s infrastructures including windbreak fencing, and management procedures. Wood shavings were provided for bedding and replenished as needed throughout the trial. On day 14 post-weaning, groups swapped pens, following Cooke et al. [15], to minimize pen effects on the experimental outcomes. For the entire experimental period, all heifers had ad libitum access to water and feed; a total mixed ration (TMR) diet formulated according to the Nutritional Requirements of Beef Cattle [23] (Table 1), which was offered once daily (~0900 h) in seven Vytelle SENSE™ (Vytelle™ SENSE, Calgary, AB, USA) feed bunks per pen. Feed bunks were inspected daily to verify continuous feed availability and to prevent feed depletion.

### 2.2. Measurements and Sampling Events

#### 2.2.1. Feed Intake and Feeding Behavior Metrics

The measurements obtained from the Vytelle SENSE™ feed bunk system included individual dry matter intake (DMI, kg/day), feeding duration (min/day), and feeding frequency (visits/day). The collected data (intake and duration) were used to calculate feeding rate (g/min) for the entire experimental period (from day 0 to 27 post-weaning), as described by González et al. [24].

#### 2.2.2. Growth Performance Metrics

On day 0, both weaning weights recorded prior to transportation were averaged to determine the pre-travel BW (kg). This value was used in combination with the shrunk BW (post-transportation) to calculate the BW loss (%) using the following Equation (1):

(1) $$BW\;loss=\left(1-\left(\frac{Shrunk\;BW}{pre-travel\;BW}\right)\right)\times 100$$

Additionally, the BW obtained pre- and post-transportation (pre-travel BW and shrunk BW, respectively) were averaged to generate the initial BW (baseline measurement). This was done to reduce variability associated with gut fill resulting from the absence of a fasting period prior to weighing [25,26]. Two additional BW assessments were conducted on days 14 (BW14, kg) and 27 (final BW, kg) post-weaning. The BW measurements obtained during the sampling events described above were used to calculate the slope of a linear regression, which determined average daily gain (ADG, kg/day) for three different periods: ADG_0–14_ (initial BW and BW14), ADG_14–27_ (BW14 and Final BW), and ADG_0–27_ (initial BW and Final BW).

In addition, the average DMI (kg/day) obtained in the initial two-weeks (from day 0 to 13; DMI_0–13_), last two-weeks (from day 14 to 27, DMI_14–27_), and the entire experimental period (from day 0 to 27, DMI_0–27_) were included in the G:F ratio calculation, using each corresponding ADG period to determine the G:F_0–13_, G:F_14–27_, and G:F_0–27_ (kg/kg).

#### 2.2.3. Stress and Health Parameters

Sensor-derived behavioral metrics (activity and rumination) were monitored in all calves (from day 0 to 27 post-weaning) using a 3-dimensional accelerometer attached to their RFID ear tags (CowManager SensOor, Agis, Harmelen, UT, the Netherlands). Data from the sensors were transmitted via a router to a computer, for storage. Each minute, the sensor continuously collected data from individual animals, classifying their behavior into one of four categories: ‘non-active’, ‘highly active’, ‘active’ or ‘rumination’, as previously described by Reynold et al. [27]. Active was obtained by summing the ‘active’ and ‘highly active’ behaviors associated with any movement, except eating and rumination. Non-active indicated no movement including rumination, eating, and activity, while rumination indicated ear movement associated with regurgitation, rumination, salivation, and swallowing of ingesta in a standing or lying position [22].

Both chute score (CS, score) [28] and chute exit speed (ES, m/s) [29] were used as behavioral indicators of reactivity at handling. The CS and ES methods were conducted immediately before and after weighing the animals, respectively, during multiple sampling occasions: twice on day 0 before treatment administration (baseline), once after the resting period, and on days 14 and 27 post-weaning. Briefly, the CS method was performed by an experienced observer by assessing the level of agitation and excitability of individual cattle that were in the squeeze chute but not restrained by the head gate, for a 4 s period. After 4 s, the observer categorized the animal on a scale of 1 to 4 where greater score values indicated greater excitability, as follow: CS (1) animal does not offer resistance, remains with head, ears, and tail relaxed; CS (2) animal shows some movement with head up and ears erect and may flick its tail occasionally; CS (3) frequent movement but not vigorous, head, ears and/or tail movements and; CS (4) abrupt and vigorous movements of the whole animal as well as the head, ear, and/or tail and may jump and/or fall. The ES method included an electronic device (FarmTek, Inc., Wylie, TX, USA) with two pairs of photoelectric cells, a chronometer and a small processor programmed that recorded the time taken by each animal to pass a known distance immediately after releasing the animal from the squeeze chute. Faster animals were considered to have more excitable reactivity at handling.

To assess acute stress at handling, saliva samples were collected twice on days 0, immediately prior to treatment administration (baseline) and after resting period as well as days 14 and 27 post-weaning, by swabbing the oral cavity with a cotton swab applicator. Samples were immediately placed in a plastic tube and frozen at −20 °C. Samples were subsequently analyzed for salivary cortisol concentration (cortisol, nmol/L) using an enzyme immunoassay kit (Salimetrics, State College, PA, USA), as previously described by Meléndez et al. [30], with intra- and inter-assay reliability 5.8% and 16.4%, respectively. Rectal temperature was collected twice on day 0 (immediately prior to transportation, used as baseline, and after the resting period), as well as days 14 and 27 using a digital thermometer (GLA Agricultural Electronics, M750 Livestock Thermometer, San Luis Obispo, CA, USA). Additionally, the same two research personnel monitored all heifers daily (from day 0 to 27 post-weaning) for clinical signs of illness or distress following the method described by Love et al. [31] and the BRD clinical sign guidelines established by the BCRC [32], removing suspected animals from the pen as needed for rectal temperature assessment and health treatments.

To assess stress and immune response, blood samples were collected from the coccygeal vein from all heifers into 6 mL vacuum tubes containing EDTA (BD Vacutainer; Becton Dickinson Co., Franklin Lakes, NJ, USA) on day 0 (immediately prior to transportation; baseline) and on days 14 and 27 post-weaning. These samples were analyzed to determine the complete blood cell count (CBC) using an Element HT5 Analyzer (Heska, Loveland, CO, USA). The CBC included measurements for red blood cells (RBC, ×10^6^/µL), hematocrit (HCT, %), hemoglobin (HGB, g/L) white blood cells (WBC, ×10^3^/µL), neutrophils (NEU, ×10^3^/µL), lymphocytes (LYM, ×10^3^/µL), monocytes (MONO, ×10^3^/µL), eosinophils (EOS, ×10^3^/µL), and platelet counts (PLT, ×10^3^/µL). The neutrophil-to-lymphocyte (N:L) ratio was calculated.

#### 2.2.4. Profitability Metrics

The profitability (adapted from Pickett et al. [20]) was calculated by first determining the net gain. This involved summing the individual BW measurements within each pen to obtain the total BW at weaning (day 0; total initial BW, kg/pen) and at the end of the trial (day 27; total final BW, kg/pen). A 4% shrink was applied to both total initial and final BW to account for excretory losses [33]. The adjusted BW values were then multiplied by a sales price of CAD$4.00/kg [34] to estimate the initial and final values per pen (CAD$/pen), as shown in the following Equation (2):

(2) $$\kappa \;Value=\left(\lambda \;BW\times \left(1-0.04\right)\right)\times CAD\$4$$

where κ represents the corresponding initial value or final value and λ represents the corresponding initial BW or final BW.

Profit per pen (CAD$/pen) over the experimental period (from day 0 to 27) was calculated as the difference between the final value and the initial value, total feed costs, and total medication costs, as described by Pickett et al. [20] using the following Equation (3):

(3) $$Profit=Final\;Value-\left(Initial\;Value+Total\;Feed\;costs+Total\;Medication\;costs\right)$$

The return-of-investment (ROI) was then calculated using the following Equation (4):

(4) $$ROI=\frac{\left(Profit\;difference\right)}{Treatment\;cost}\times 100$$

where ROI indicates the return of investment; profit difference refers to the difference in profit obtained between the two treatment groups; and treatment cost represents the total cost of mBAS.

### 2.3. Data Management and Statistical Analysis

A single heifer (CT group) exhibited weight loss (−0.08 kg/day) between days 14 and 27; therefore, its growth performance data for day 27, and respective ADG calculation were excluded from the final dataset. Feed intake and feeding behavior recorded from all heifers on day 27 were included in the dataset up to the handling event (10:00 AM). Then, the final intake and feeding behavior dataset were summarized on a daily and weekly basis (average data obtained from day 0 to 6, 7 to 13, 14 to 20, and 21 to 27 representing weeks 1, 2, 3, and 4, respectively). Sensor-derived behavioral data (activity and rumination) were cleaned by including only records after the completion of all handling procedures in both treatment groups (day 0; starting at 4:00 PM). Similarly, data recorded during the two-hour handling period (for both groups) on day 14 were removed. On day 27, sensor-derived data was collected until 10:00 AM for both groups. The final activity and rumination dataset were summarized on a daily and weekly basis (as described above). Additionally, for any animals suspected of illness or distress that were removed from the pen for rectal temperature assessment, the timeframe during which they were handled was noted, and corresponding sensor data were excluded from the final dataset. Prior to statistical analysis, all activity and rumination data were converted to percentages; each value was divided by the number of minutes in 24 h (1440 min) and multiplied by 100.

Data analyses were carried out using SAS^®^ (version 9.4) [35]. Animals were considered as experimental units, as groups were rotated between pens. Generalized linear mixed-effects models (SAS PROC GLIMMIX) were used in all analyses described below. For feed intake, feeding behavior, sensor-derived behavioral metrics, CBC, ES, cortisol, and rectal temperature, the statistical model included treatment, day, and their interaction as fixed effects. Animal was included as a random effect, and sampling days were treated as repeated measures using the RANDOM statement. Moreover, for CS, ES, cortisol, and temperature obtained prior to treatment administration (day 0), the initial BW (calculated as the average of BW recorded pre- and post-transportation) served as the covariate. Additionally, for CBC metrics, ES, rectal temperature and cortisol, all pre-treatment measurements of the dependent variables were included as covariates in the model. The optimal distribution was selected based on the Bayesian Information Criterion (BIC), followed by the evaluation of covariance structures, with the one yielding the lowest Schwarz’s BIC selected for the final analysis.

For shrunk BW, BW14, BW27, BW loss, as well as ADG, and G:F, treatment was included as a fixed effect. For shrunk BW, the initial BW was used as a covariate. For BW14, BW27, ADG, and G:F, the average of the two weaning weights (pre- and post-transport) served as the covariate.

Result values correspond to non-transformed least square means; however, the SEM and the *p*-values correspond to GLIMMIX analysis using napierian log transformation. Least square means differences were determined using the PDIFF option in SAS. A post hoc test (Tukey) was employed to compare the adjusted means. Main effects were considered significant at *p* ≤ 0.05 and a tendency at 0.05 < *p* ≤ 0.10.

## 3. Results

### 3.1. Feed Intake and Feeding Behavior Metrics

**Figure 1:**
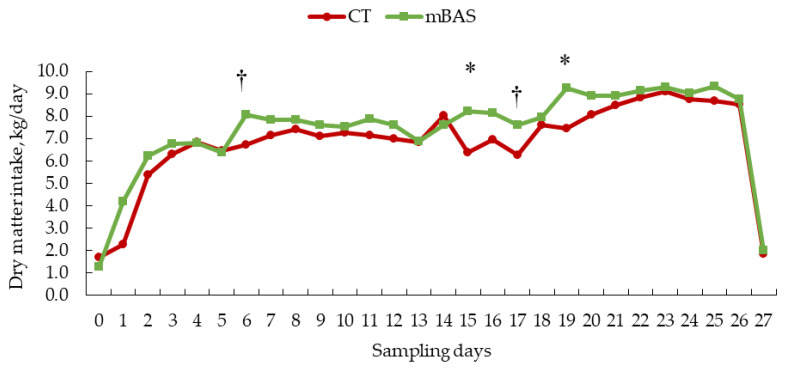
Least square means of dry matter intake (min/day) of Angus-influenced heifers housed in feedlot pens over a 28-day period ^1^, following treatment with either 10 mL maternal appeasing substance (mBAS; *n* = 11) or 10 mL water, used as control group (CT; *n* = 11) at weaning, immediately prior to a 1 h transportation. ^1^ On day 27, dry matter intake data was collected until 10:00 AM for both treatment groups. * Symbol denotes means that differ significantly at the *p* < 0.05 level. † Symbol denotes a tendency of significance at 0.05 < *p* ≤ 0.10 level. Days 0, 14, and 27 represent the handling event days.

**Figure 2:**
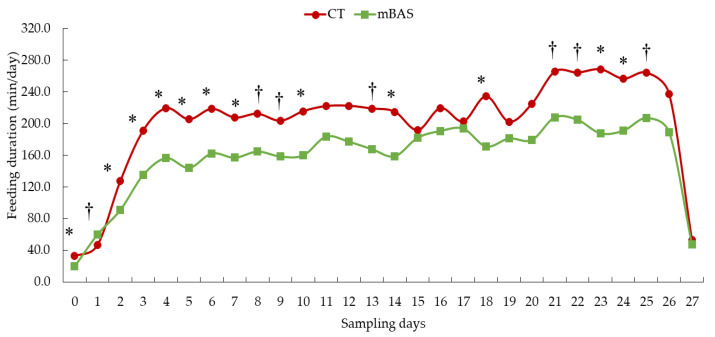
Least square means of feeding duration (min/day) of Angus-influenced heifers housed in feedlot pens over a 28-day period ^1^, following treatment with either 10 mL maternal appeasing substance (mBAS; *n* = 11) or 10 mL water, used as control group (CT; *n* = 11) at weaning, immediately prior to a 1 h transportation. ^1^ On day 27, feeding duration data was collected until 10:00 AM for both treatment groups. * Symbol denotes means that differ significantly at the *p* < 0.05 level. † Symbol denotes a tendency of significance at 0.05 < *p* ≤ 0.10 level. Days 0, 14, and 27 represent the handling event days.

**Figure 3:**
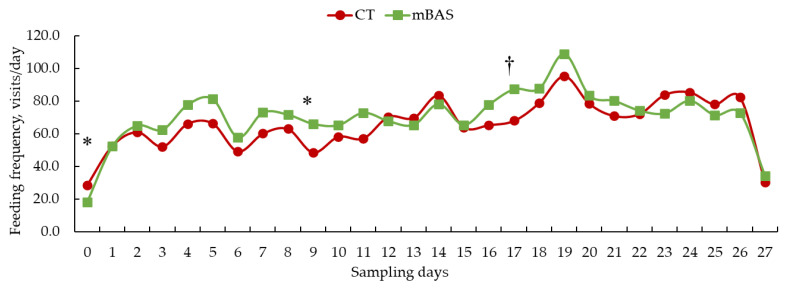
Least square means of feeding frequency (visits/day) of Angus-influenced heifers housed in feedlot pens over a 28-day period ^1^, following treatment with either 10 mL maternal appeasing substance (mBAS; *n* = 11) or 10 mL water, used as control group (CT; *n* = 11) at weaning, immediately prior to a 1 h transportation. ^1^ On day 27, feeding frequency data was collected until 10:00 AM for both treatment groups. * Symbol denotes means that differ significantly at the *p* < 0.05 level. † Symbol denotes a tendency of significance at 0.05 < *p* ≤ 0.10 level. Days 0, 14, and 27 represent the handling event days.

Results for feed intake and feeding behavior metrics are presented in Table 2 as well as Figure 1, Figure 2 and Figure 3. When data was evaluated on daily basis, a tendency (treatment × day; *p* = 0.09) interaction was found for dry matter intake. Likewise, a tendency (treatment × day; *p* = 0.08) was observed for feeding duration while a significant interaction (treatment × day; *p* = 0.02) was detected for feeding frequency, and a significant effect (treatment effect; *p* < 0.01) was found for feeding rate.

During the first two weeks post-weaning, heifers administered mBAS exhibited a tendency of greater dry matter intake on day 6 (*p* = 0.06) than CT heifers (Figure 1). In the final two weeks of the trial, mBAS heifers had greater (*p* < 0.05) intake on days 15 and 19 and a tendency of greater intake on day 17 (*p* = 0.06) post-weaning. No differences (*p* > 0.10) were observed on the remaining days of the experimental period (Figure 1).

Shorter (*p* < 0.001) feeding durations were observed on day 0, as well as from days 2 to 7 (*p* ≤ 0.05), and on days 10, 14, 18, 23, and 24 post-treatment (*p* < 0.05) than CT heifers (Figure 1). The mBAS heifers tended to spend more time (*p* = 0.07) feeding on day 1 but less time on several later days (8, 9, 13, and 21; *p* = 0.08) as well as on day 22 (*p* = 0.07) and day 25 (*p* = 0.09) compared with CT heifers (Figure 2). No differences (*p* > 0.10) were observed on days 11, 12, 15, 16, 17, 19, 20, 26, or 27 post-treatment administration (Figure 2).

Conversely, mBAS-treated heifers exhibited significantly lower (*p* < 0.001) feeding frequency on day 0, but greater (*p* < 0.05) on day 9, with a trend (*p* = 0.07) for increased frequency on day 17 than CT heifers (Figure 3). No differences (*p* > 0.10) were observed on the remaining days of the experimental period (Figure 3).

Additionally, the mBAS-treated heifers had greater (treatment effect; *p* < 0.01) feeding ratio than CT heifers (Table 2).

When data were analyzed on a weekly basis, a tendency of treatment × day (*p* = 0.06) interaction was observed for dry matter intake (Table 2); however, differences were primarily within rather than between treatments (*p* > 0.10. Significant treatment × day (*p* < 0.05) interactions were detected for feeding duration, feeding frequency, and feeding rate (Table 2). The mBAS-treated heifers had a shorter (*p* < 0.001) feeding duration during week 1 and showed a tendency for shorter (*p* = 0.06) duration in week 2 (112.7 and 178.2 ± 0.06 min/day, respectively) than CT heifers (154.8 and 206.1 ± 0.05 min/day, for weeks 1 and 2, respectively), with no differences (*p* > 0.10) observed in weeks 3 or 4. Differences in feeding frequencies were detected within treatment groups rather than between groups (*p* > 0.10). On the other hand, mBAS-treated heifers had a significantly greater (*p* < 0.001) feeding ratio during week 1 compared with CT heifers (52.7 and 40.7 ± 0.04 min/week, respectively), with no differences (*p* > 0.10) observed in weeks 2, 3, or 4 (*p* > 0.10).

### 3.2. Growth Performance Metrics

Results for growth performance metrics are presented in Table 3. As per the study design, no difference (*p* > 0.10) was found for initial BW. Although no significant (*p* > 0.10) differences were found for shrunk BW, BW loss, BW 14, or initial ADG, mBAS heifers had greater (*p* ≤ 0.05) final BW, final ADG, and total ADG compared to CT heifers. Likewise, no differences (*p* > 0.10) were found for initial G:F; however, greater (*p* < 0.05) final and total G:F ratio were observed for mBAS compared to CT heifers.

### 3.3. Stress and Health Parameters

**Figure 4:**
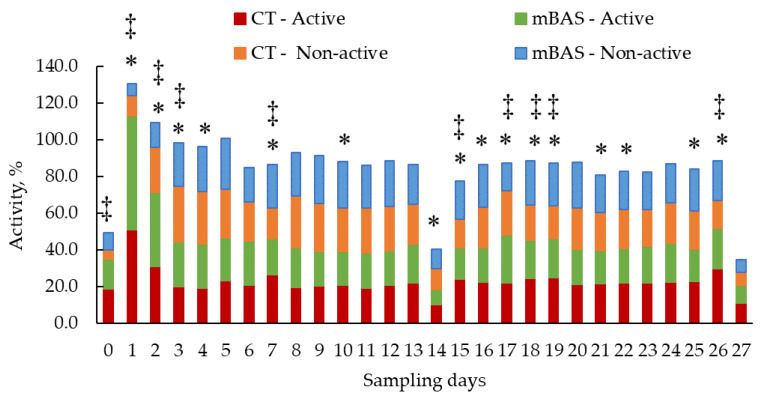
Least square means for sensor-derived behavioral metrics (activity; active and non-active) of Angus-influenced heifers housed in feedlot pens over a 28-day period ^1^, following treatment with either 10 mL maternal appeasing substance (mBAS; *n* = 11) or 10 mL water, used as control group (CT; *n* = 11) at weaning, immediately prior to a 1 h transportation. ^1^ On day 27, activity data was collected until 10:00 AM for both treatment groups. * Symbol denotes (active) means that differ significantly at the *p* ≤ 0.05 level. ‡ Symbol denotes (non-active) means that differ significantly at the *p* ≤ 0.05 level.

**Figure 5:**
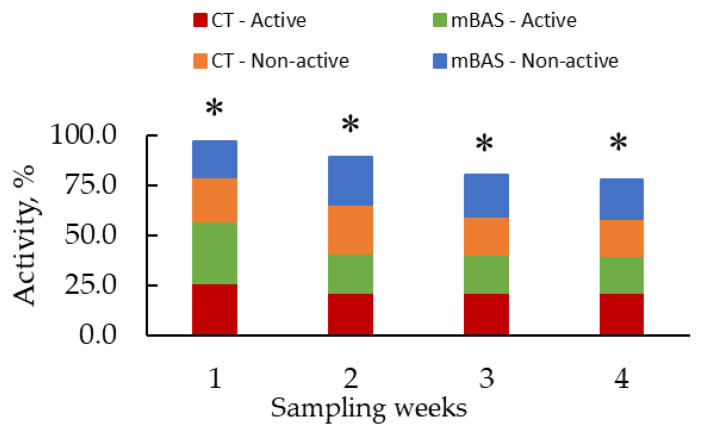
Least square means for sensor-derived behavioral metrics (activity; active and non-active) of Angus-influenced heifers housed in feedlot pens over a four-week period ^1^, following treatment with either 10 mL maternal appeasing substance (mBAS; *n* = 11) or 10 mL water, used as control group (CT; *n* = 11) at weaning, immediately prior to a 1 h transportation. ^1^ On day 27, activity data was collected until 10:00 AM for both treatment groups. * Symbol denotes (active) means that differ significantly at the *p* < 0.05 level.

**Figure 6:**
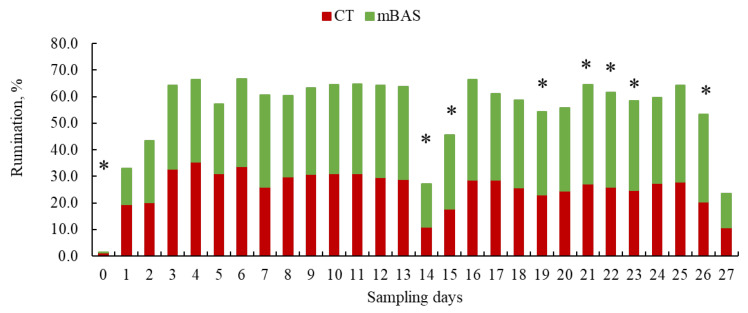
Least square means for sensor-derived behavioral metrics (rumination) of Angus-influenced heifers housed in feedlot pens over a 28-day period ^1^, following treatment with either 10 mL maternal appeasing substance (mBAS; *n* = 11) or 10 mL water, used as control group (CT; *n* = 11) at weaning, immediately prior to a 1 h transportation. ^1^ On day 27, rumination data was collected until 10:00 AM for both treatment groups. * Symbol denotes means that differ significantly at the *p* < 0.05 level.

**Figure 7:**
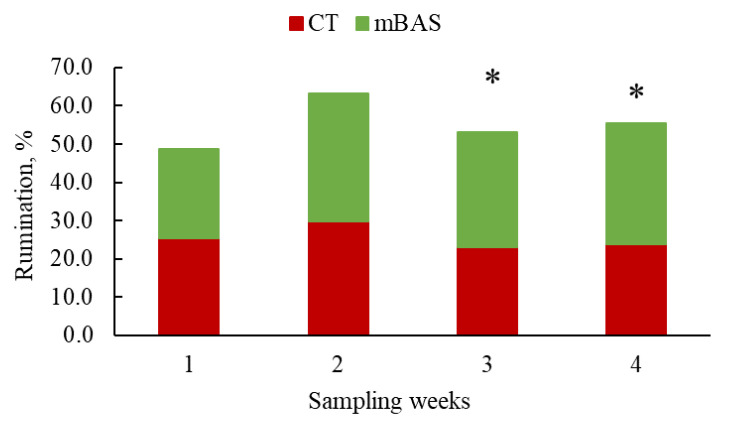
Least square means for sensor-derived behavioral metrics (rumination) of Angus-influenced heifers housed in feedlot pens over a four-week period ^1^, following treatment with either 10 mL maternal appeasing substance (mBAS; *n* = 11) or 10 mL water, used as control group (CT; *n* = 11) at weaning, immediately prior to a 1 h transportation. ^1^ On day 27, rumination data was collected until 10:00 AM for both treatment groups. * Symbol denotes means that differ significantly at the *p* < 0.05 level.

Results for sensor-derived behavioral metrics (activity and rumination) are presented in Table 4 and in Figure 4, Figure 5, Figure 6 and Figure 7, while reactivity at handling metrics, cortisol, rectal temperature, and CBC metrics are presented in Table 5. Significant (treatment × day or week; *p* < 0.01) interactions were observed when data were evaluated using both daily and weekly basis sensor-derived behavioral metrics.

When data was evaluated on daily basis, mBAS heifers were more active (*p* < 0.01) than CT heifers early (from day 1 to 4, and day 17) but less active (*p* < 0.05) on several later days (days 7, 10, 14, 15, 16, 18, 19, 21, 22, 25, and 26) (Figure 4). No differences (*p* > 0.10) were observed between treatment groups on the remaining days (Figure 4). Conversely, mBAS heifers were less (*p* < 0.05) non-active from days 1 to 3 and day 17, but more (*p* < 0.05) non-active on days 0, 7, 15, 18, 19, and 26 compared with CT heifers (Figure 4). Again, no differences (*p* > 0.10) were observed for non-active behavior on the remaining days (Figure 4).

When data was analyzed on a weekly basis, mBAS heifers displayed a greater (*p* < 0.01) percentage of active behavior on week 1, but lower percentage of active behavior (*p* ≤ 0.05) on weeks 2, 3, and 4 than CT heifers (Figure 5). For non-active behavior, mBAS heifers had lower (*p* < 0.05) percentage active behavior on week 1 than CT heifers, but no differences (*p* > 0.10) were detected on weeks 2, 3, or 4 (Figure 5).

For rumination behavior, mBAS heifers exhibited lower (*p* < 0.01) rumination behavior percentages on day 0, but greater (*p* < 0.05) percentages on days 14, 15, 19, as well as from days 21 to 23, and on day 26 compared to CT heifers (Figure 6). Additionally, mBAS heifers tended to have greater (*p* = 0.07) percentages on days 7 and 25, and a tendency for greater (*p* = 0.06) rumination behavior percentages on day 16 than CT heifers (Figure 6).

When data was summarized on weekly basis, mBAS heifers exhibited greater (*p* < 0.05) percentages of rumination on weeks 3 and 4 (30.2 and 31.7%, respectively) compared to CT heifers (23.0 and 23.8%, respectively), but no differences (*p* > 0.10) were found on weeks 1 or 2 (Figure 7).

No significant differences (*p* > 0.10) were observed in reactivity at handling parameters (CS or ES), salivary cortisol, or rectal temperature between treatment groups (Table 5). However, significant (treatment × day; *p* < 0.05) interactions were observed for three out of the ten CBC parameters (RBC, HCT, and LYM) and tendencies (treatment effect; *p* ≤ 0.09) were found for N:L ratio and PLT (Table 5). No differences (*p* > 0.10) were found for HGB, WBC, NEU, MONO, or EOS between treatment groups (Table 5).

For the CBC parameters, although a significant (treatment × day; *p* < 0.05) interaction was observed for RBC (Table 5), pairwise differences occurred within treatment groups rather than between them (*p* > 0.10). The mBAS heifers exhibited greater (*p* < 0.01) HCT percentage on day 27 compared to CT heifers (36.2 and 33.6 ± 0.02%, respectively), but no differences (*p* > 0.10) were observed on day 14. Conversely, lower (*p* < 0.05) LYM counts were observed in mBAS heifers on day 14 compared to CT heifers (5.9 and 6.6 ± 0.03 ×10^3^/µL, respectively), with no differences (*p* > 0.10) detected on day 27. Similarly, a tendency for lower (*p* = 0.09) N:L ratio and a tendency for greater (*p* = 0.08) PLT counts were observed in mBAS heifers compared with CT heifers for the entire experimental period (Table 5).

One animal from each treatment had to be treated more than once (days 0 and 14) due to rectal temperature greater than 40 °C. No incidence of mortality was observed during the experimental period.

### 3.4. Profitability Metrics

Results for total profitability metrics are shown in Table 6. When using net gain metrics for the ROI calculation, our findings indicate that for every CAD$1 spent on mBAS treatment, the return is approximately CAD$11.74 in profit, based on liveweight-adjusted mBAS treatment costs.

## 4. Discussion

Weaning is one of the most stressful events in a beef calf’s life, with important consequences for health, welfare, and subsequent growth [2]. During this event, calves face multiple concurrent stressors that can impair immunity, reduce feed intake, and hinder growth [1,2,7], with the severity of responses varying according to individual stress resilience [36,37]. This study evaluated whether a single pre-transport administration of 10 mL mBAS at weaning could reduce transport-induced weight loss and improve feedlot adaptation in Angus-influenced heifers. Although mBAS had positive effects on certain indicators of health, stress, and growth, the overall outcomes did not fully meet initial expectations.

As anticipated from the study design, no differences in initial body weight were observed between treatment groups. However, a delayed positive effect of mBAS emerged, with treated heifers demonstrating superior growth performance during the third- and fourth-weeks post-weaning (Final BW, ADG_14–27_, and ADG_0–27_) compared to their water-treated counterparts. In summary, by the end of the 28-day trial, mBAS-treated heifers were 3.4% heavier (+12.3 kg) than controls. They also exhibited a 57.1% greater daily gain in weeks three and four (+800 g/day) and a 27.8% higher overall ADG (+500 g/day). These results are partially consistent with previous findings where mBAS was administered prior to loading or at feedlot entry. In Nellore bulls, weight gains were observed during the adaptation period (up to 19 days) when mBAS was applied at either time point [17]. However, these advantages were not sustained through 60 days of the feeding period, and overall improvements up to day 108 were only maintained when mBAS was administered prior to loading [17]. Likewise *Bos taurus* × *Bos indicus* calves showed improved growth rates up to 28 days when mBAS was administered at weaning [18]. However, these benefits were not sustained beyond this period, with no effects detected by day 42 [18]. Other studies applying mBAS at feedlot entry have also yielded contradictory findings. Picket et al. [20] found no effects on growth performance up to 60 days, whereas Colombo et al. [19] reported no effects on body weight at days 7, 17, 31, or 45 but did observe overall weight gain across the entire feeding period. These discrepancies between the current and previous studies [17,18,19,20] remain unclear but suggest that the efficacy of mBAS may be context-dependent, influenced by factors such as time of administration, study length, or breed-specific stress responses, and warrant further investigation.

Notably, the enhanced growth observed in the current study was not driven by differences in dry matter intake. Overall, both groups consumed similar amounts of feed, averaging approximately 7.0 kg/head/day, consistent with previous findings for feedlot cattle during the first four weeks post-weaning [38]. Instead, the improved performance likely reflects greater feed efficiency across the entire trial period, but particularly evident during the latter half of the study. These findings align with those reported by Colombo et al. [19] who found a greater gain-to-feed ratio over a 45-day period in Angus-influenced calves treated with mBAS at feedlot entry compared with untreated controls. On the other hand, Schubach et al. [18] reported no effects on feed efficiency over 42 days in *Bos taurus* × *Bos indicus* calves when mBAS was applied at weaning. In contrast to the current study, Fonseca et al. [17] reported a greater gain-to-feed ratio during the 19-day adaptation period in Nellore bulls when mBAS was administered at either prior to loading or feedlot entry, but long-term improvements over the 108-day feeding period were evident only when administered prior to loading.

The enhanced efficiency observed in the current study may have resulted from mBAS-mediated attenuation of stress-induced activation of the hypothalamic–pituitary–adrenal (HPA) axis, reducing secretion of adrenocorticotropic hormone (ACTH) and cortisol [39,40]. By diminishing the physiological stress response, mBAS likely reduces the metabolic costs of stress, supporting more efficient nutrient utilization and contributing to the growth advantage observed. Other mechanisms may also contribute, including potential effects on nutrient digestibility, rumen function [41], behavioral circadian rhythms [42], or even factors related to experimental conditions. The previously cited studies used larger groups (>40 animals per treatment), exposing cattle to greater disease and social stress. By contrast, the smaller groups in the present study likely reduced stress, contributing to improved feed efficiency. Therefore, the outcomes observed in this study should be interpreted with caution, as they may not fully reflect the conditions or results expected in typical commercial feedlot environments.

Given the lack of effects on shrunk body weight and percentage of body weight loss (both groups lost 1.4% of BW), two possible explanations can be considered: (1) although transport conditions were carefully standardized between groups, potential confounding factors such as interior ventilation, vibration frequency, noise, and light levels, could have influenced the outcomes and; (2) the transportation conditions in this trial may not have been sufficient to elicit a measurable stress response capable of triggering acute metabolic losses. Heifers were transported approximately 84 km (1 h), and all handling procedures, including loading, transport, unloading, resting, and post-transport weighing and sampling, were conducted calmly by experienced handlers who were familiar to the animals. The entire process was completed within about three hours per group.

Previous research has shown that salivary cortisol concentrations in calves rise within 20 min following artificial ACTH stimulation (as a proxy of stress response) and typically return to baseline in approximately three hours [43]. Since our sampling period fell within this expected window, we initially hypothesized that treatment differences in cortisol would be detectable. However, it is possible that a transient cortisol peak occurred outside the specific sampling points, or alternatively, that the relatively mild transport and handling conditions limited HPA axis activation to levels undetectable in salivary samples. Cortisol, a key stress hormone released via HPA axis activation, supports short-term adaptation by mobilizing energy through gluconeogenesis [13,39,44]. However, prolonged elevation can suppress immunity, reduce growth, and compromise animal health and performance [39,44]. Our findings in salivary cortisol align with previous studies [15,17] reporting no differences in serum (or plasma) cortisol concentrations between cattle that received or not a single treatment of mBAS prior to loading or at weaning. The lack of physiological differences was consistent with the absence of behavioral differences at handling, as assessed through chute score and exit speed. In contrast, stress-mitigating effects of mBAS have been reported in other contexts. Colombo et al. [19] observed reduced plasma cortisol on days 7 and 17 relative to feedlot arrival, but not on days 31 or 45. Schubach et al. [18], found no effects of mBAS on plasma cortisol over 42 days; however, they reported a tendency for lower exit speed by day 7, a significant exit speed reduction by day 14, and decreased hair cortisol concentrations also on day 14. Unlike serum, plasma, or salivary cortisol, which reflects acute fluctuations, hair cortisol represents cumulative HPA axis activity over time [45]. Taken together, these findings suggest that the effects of mBAS on cortisol measures are most evident during the early phase of feedlot adaptation, consistent with its effectiveness for approximately two weeks post-application, with no sustained benefits observed beyond this period.

Although mBAS heifers demonstrated indications of greater dry matter intake than controls on days 6, 15, 17, and 19 of the 28-day period, the absence of consistent overall differences aligns with previous research, where mBAS administration prior to loading or at feedlot entry in *Bos indicus* or *Bos taurus* cattle produced inconsistent or negligible effects on intake when evaluated at the pen level [17,18,19,20]. These inconsistencies may be attributed to differences in social dynamics, metabolic regulation, or breed-specific temperament. In addition, variation in feed intake measurement methodology, especially between individual- versus pen-level data, may further contribute to uneven outcomes. To the best of our knowledge, this is the first study to evaluate mBAS effects using individual feed intake data from feedlot cattle in North America, allowing for a more precise assessment of feeding behavior.

Interestingly, during the first two weeks, treated heifers spent less time at the feed bunk but consumed feed at a faster rate during week 1, despite only slight differences between treatments in bunk visits. This feeding pattern mirrors findings by Proudfoot et al. [46], who reported that multiparous dairy cows in competitive environments, such as the initial feeding period, increased their eating rate to maintain intake while reducing time at the bunk. Similarly, mBAS-treated heifers appeared to adopt a feeding strategy characterized by shorter, more purposeful bouts at the bunk, thereby limiting energy expenditure and competition while supporting recovery and adjustment to the feedlot environment. A previous study also reported that mBAS-treated calves spent more time in physical activity such as engaging in social behaviors (e.g., mounting) compared with untreated calves, particularly during the first two days in the feedlot, a behavior associated not only with reproductive activity but also with the establishment of social hierarchy [18].

The initial increase in activity may be attributed to either (1) stress associated with maternal separation or (2) exploratory motivation to interact with novel elements of the environment, such as the feed bunk [18,47]. In contrast, the subsequent decline may indicates calmer behavior, as also reported by Schubach et al. [18]. In their study, mBAS treated calves tended to display more allogrooming and exhibited increased physical activity on day 1 compared with controls, but no differences were observed on subsequent days (up to day 41). Although the mechanisms remain unclear, this shift observed in the current study likely reflects reduced stress reactivity and more effective allocation of energy toward nutrient utilization, ultimately contributing to improved feed efficiency [48]. Given the known 15-day efficacy of mBAS [18], these findings suggest that the product may help calves transition more smoothly during the critical early adaptation period by modulating feeding dynamics and behavioral responses [17,18].

Consistent with this, mBAS-treated heifers ruminated more during the third- and fourth-weeks post-weaning. Because stress disrupts feeding behavior and reduces rumination [49,50] the increase observed here suggests improved adaptation. Greater rumination is linked to enhanced weight gain and feed efficiency [49,51], which may partially explain the improved growth performance observed in the latter phase of the trial. The extended rumination observed in mBAS-treated heifers may also indicate improved fiber digestibility and rumen development [41], supported by higher saliva flow, stable rumen pH, and greater microbial activity [52,53]. Collectively, these changes indicate that mBAS-treated heifers adopted a coping strategy that improved nutrient utilization and growth without increasing feed intake. The observation that mBAS-treated heifers spent less time ruminating on day 0 compared to controls contradicts our initial hypothesis and remains unclear; however, since this difference was observed solely on day 0 and represented a difference of 0.9%, it may lack biological significance. To our knowledge, this is the first study to evaluate rumination as a stress-related behavioral indicator of mBAS treatment in feedlot calves, highlighting the need for further research on its role in long-term welfare and adaptation.

Although the stressors experienced by the animals in this study do not fully replicate those encountered in commercial Canadian feedlots, our findings suggest that mBAS treatment may help mitigate stress and support immune function during challenging events such as weaning. At feedlot entry, the acute phase response primes the body to defend against potential injury or infection [54]. This was reflected in lower LYM counts on day 14 in mBAS-treated heifers compared with controls. Such a reduction in LYM likely represents an alternative pattern of immune modulation, suggesting that mBAS heifers adapted to stress by mobilizing defenses more efficiently without inducing excessive or prolonged immunosuppression. Platelets, in addition to their central role in clot formation, contribute to immune regulation, inflammation, and pathogen defense [55]. Stress can influence PLT activation and numbers [56], and the elevated PLT counts observed in mBAS heifers throughout the trial may indicate a more robust capacity to respond to injury or infection during stressful transitions. Collectively, these findings support the concept that mBAS modulates stress pathways in ways that enhance physiological resilience.

An increase in HCT usually indicates hemoconcentration caused by dehydration or stress-related plasma volume shifts [54]. However, given that greater HCT was observed only on day 27, these findings likely lack biological meaning, especially considering that the acute phase response generally returns to baseline within 4 weeks post-feedlot receiving [57]. Similarly, the minimal difference in N:L ratio between groups (+0.1) may not represent a meaningful biological effect. Previous studies have shown that mBAS treatment in feedlot cattle can reduce stress responses using acute-phase proteins (APPs) such as haptoglobin, enhance immune function [15] and vaccine responses [18], and enable earlier detection and treatment of bovine respiratory disease [20]. Our findings support this evidence, suggesting that mBAS may help mitigate stress-induced immune alterations. However, the absence of rectal temperature differences and the corresponding lack of changes in other leukocyte parameters (WBC, NEU) indicate that further investigation is warranted.

Although our findings are consistent with those of Picket et al. [20], who reported similar benefits in 120 Angus-influenced calves over 60 days, the exceptionally high ROI of 1174% observed here should be interpreted cautiously. While mathematically accurate, this estimate may have been inflated by the small sample size and short trial duration. Thus, we consider this outcome an indication of the potential for positive economic returns rather than a definitive estimate of sustained profitability.

Despite these encouraging results, several limitations and potential sources of bias should be acknowledged. For health outcomes, the relatively low disease challenge in this study may have limited the ability to detect treatment effects on morbidity and immune responses. For stress indicators, variability in salivary cortisol combined with the limited number of sampling points may have underestimated acute differences between groups. For growth outcomes, the small sample size may have introduced bias and reduces the generalizability of the findings to larger, commercial-scale feedlot operations. In addition, calculating profitability at the pen level with only one pen per treatment is a further limitation that may have influenced ROI estimates. Finally, environmental and management factors, such as transport conditions, could also have contributed to variation in behavioral and performance responses. Future research should include larger sample sizes, true pen replication, longer monitoring periods, and more diverse management systems to more robustly evaluate the efficacy of mBAS under commercial feedlot conditions.

## 5. Conclusions

This study indicates that while mBAS did not reduce transportation-related weight loss or consistently affect feed intake, it appeared to enhance the adaptability of weaned beef heifers to the feedlot environment. By modulating feeding behavior, supporting growth performance, improving selected immune measures, and influencing activity and rumination, mBAS was associated with improved feed efficiency. However, further research is needed to determine whether these short-term responses translate into lasting effects on metabolic adaptation, immune resilience, and overall profitability.

Beyond the immediate production outcomes, the findings are relevant to the beef industry, where reducing stress at weaning remains a major challenge for both animal welfare and economic sustainability. Importantly, these results provide a preliminary basis for considering mBAS as part of broader herd health and management programs, including stress reduction during routine handling procedures. Nevertheless, confirmatory studies are recommended to establish long-term impacts, evaluate economic returns under diverse feedlot conditions, and assess applicability across breeds, ages, and management systems.

## Figures and Tables

**Table 1:** Composition and nutritional profile of the total mixed ration offered for ad libitum consumption to Angus-influenced heifers housed in feedlot pens over a 28-day period.

Item	Value
Dry-matter, %	56.1
*Composition*, *dry matter basis*	
Wheat silage, %	78.3
Barley grain, %	15.8
Mineral mix ^1^, %	5.9
*Nutritional profile* ^2^, *dry matter basis*	
Net energy for maintenance, Mcal/kg	0.80
Net energy for gain, Mcal/kg	0.51
Total digestible nutrients, %	70.0
Acid detergent fiber, %	24.5
Neutral detergent fiber, %	37.5
Crude protein, %	11.3

^1^ Containing 0.61% calcium, 0.26% phosphorus, 0.17% magnesium, 1.19% potassium, 0.21% sodium, 295.00 ppm iron, 104.00 ppm manganese, 91.00 zinc, 30.00 copper (BE 32:12 Grower Supplement M440—MSH; Bullseyefeeds^®^, Strathmore, AB, Canada). ^2^ Wet chemistry procedures conducted by the Cumberland Valley Analytical Services, Waynesboro, PA, USA.

**Table 2:** Least square means (±SEM) of dry matter intake and feeding duration, feeding frequency, and feeding rate of Angus-influenced heifers housed in feedlot pens over a 28-day period receiving (mBAS) or not (CT) a maternal bovine appeasing substance at weaning, immediately prior to a 1 h transportation.

Item ^1^	Treatments ^2^		SEM	*p*-Value ^3^		
	mBAS	CT		T	D	T × D
*Daily basis*						
Dry matter intake, kg/day	6.9	6.4	0.05	0.473	<0.001	0.093
Feeding duration, min/day	144.9	183.5	0.06	0.004	<0.001	0.079
Feeding frequency, visits/day	67.7	63.6	0.07	0.515	<0.001	0.015
Feeding rate, g/min/day	43.1 ^a^	37.7 ^b^	0.03	0.009	<0.001	0.368
*Weekly basis*						
Dry matter intake, kg/day	6.9	7.3	0.05	0.480	<0.001	0.059
Feeding duration, min/day	168.4	191.7	0.05	0.073	<0.001	<0.001
Feeding frequency, visits/day	73.7	65.4	0.10	0.444	<0.001	0.025
Feeding rate, g/min/day	42.5	38.9	0.04	0.147	<0.001	<0.001

^a,b^ Different superscript letters indicate significant differences (*p* < 0.05), while 0.05 < *p* ≤ 0.10 was considered a tendency. ^1^ The values correspond to non-transformed means; however, SEM and *p*-values correspond to GLIMMIX analysis using napierian log transformation. ^2^ Treatments were applied topically (5 mL/site to the nuchal area and muzzle). mBAS = 10 mL maternal appeasing substance (*n* = 11); CT = 10 mL water, used as control (*n* = 11). ^3^ T: treatment; D: day.

**Table 3:** Least square means (±SEM) of growth performance metrics of Angus-influenced heifers housed in feedlot pens over a 28-day period receiving (mBAS) or not (CT) a maternal bovine appeasing substance at weaning, immediately prior to a 1 h transportation.

Item ^1^	Treatments ^2^		SEM	*p*-Value
	mBAS	CT		
Initial BW, kg	314.3	317.5	11.94	0.853
Shrunk BW, kg	310.9	311.4	1.04	0.757
BW loss, %	1.4	1.4	0.20	0.908
BW 14, kg	346.6	344.6	2.44	0.578
Final BW, kg	364.2	351.9	4.24	0.048
ADG_0–14_, kg/day	2.2	2.1	0.17	0.578
ADG_14–27_, kg/day	1.4 ^a^	0.6 ^b^	0.23	0.050
ADG_0–27_, kg/day	1.8	1.3	0.16	0.048
G:F_0–14_ ^1^, kg/kg	0.34	0.30	0.07	0.219
G:F_14–27_ ^1^, kg/kg	0.15 ^a^	0.07 ^b^	0.20	0.032
G:F_0–27_ ^1^, kg/kg	0.24 ^a^	0.16 ^b^	0.10	0.002

^a,b^ Different superscript letters indicate significant differences (*p* < 0.05), while 0.05 < *p* ≤ 0.10 was considered a tendency. ^1^ The values correspond to non-transformed means; however, SEM and *p*-values correspond to GLIMMIX analysis using napierian log transformation. ^2^ Treatments were applied topically (5 mL/site to the nuchal area and muzzle). mBAS = 10 mL maternal appeasing substance (*n* = 11); CT = 10 mL water, used as control (*n* = 11).

**Table 4:** Least square means (±SEM) of sensor-derived behavioral metrics of Angus-influenced heifers housed in feedlot pens over a 28-day period receiving (mBAS) or not (CT) a maternal bovine appeasing substance at weaning, immediately prior to a 1 h transportation.

Item ^1^	Treatments ^2^		SEM	*p*-Value ^3^		
	mBAS	CT		T	D	T × D
*Daily basis*						
Active, %	20.4	21.8	0.02	0.043	<0.001	<0.001
Non-active, %	19.2	19.1	0.05	0.910	<0.001	<0.001
Rumination, %	25.2	22.6	0.09	0.405	<0.001	<0.001
*Weekly basis*						
Active, %	21.4	22.7	0.02	0.055	<0.001	0.002
Non-active, %	20.5	20.5	0.05	0.957	<0.001	0.001
Rumination, %	29.4	25.4	0.08	0.215	<0.001	0.002

^1^ The values correspond to non-transformed means; however, SEM and *p*-values correspond to GLIMMIX analysis using napierian log transformation. ^2^ Treatments were applied topically (5 mL/site to the nuchal area and muzzle). mBAS = 10 mL maternal appeasing substance (*n* = 11); CT = 10 mL water, used as control (*n* = 11). ^3^ T: treatment; D: day. Main effect and interaction were considered significantly different at *p* < 0.05, while 0.05 < *p* ≤ 0.10 was considered a tendency.

**Table 5:** Least square means (±SEM) of reactivity at handling and health metrics of Angus-influenced heifers housed in feedlot pens over a 28-day period receiving (mBAS) or not (CT) a maternal bovine appeasing substance at weaning, immediately prior to a 1 h transportation.

Item ^1^	Treatments ^3^		SEM	*p*-Value ^4^		
	mBAS	CT		T	D	T × D
CS ^2^ score	1.7	2.0	0.13	0.357	0.005	0.405
ES, m/s	1.1	1.0	0.06	0.870	0.009	0.946
Cortisol, nmol/L	2.6	3.2	0.10	0.194	0.965	0.895
Temperature, °C	39.3	39.2	0.09	0.405	<0.001	0.502
RBC ^2^, ×10^6^/µL	9.8	9.7	0.02	0.680	<0.001	0.019
HCT ^2^, %	36.4	34.6	0.02	0.041	0.001	0.015
HGB ^2^, g/L	124.1	120.2	0.02	0.162	0.155	0.107
WBC ^2^, ×10^3^/µL	8.9	9.6	0.03	0.206	0.016	0.275
NEU ^2^, ×10^3^/µL	2.0	2.4	0.08	0.100	0.001	0.599
LYM ^2^, ×10^3^/µL	6.2	6.4	0.03	0.511	0.510	0.012
N:L ^2^, ratio	0.3	0.4	0.04	0.085	<0.001	0.234
MONO ^2^, ×10^3^/µL	0.2	0.2	0.09	0.743	0.019	0.149
EOS ^2^, ×10^3^/µL	0.1	0.2	0.01	0.413	0.015	0.381
PLT ^2^, ×10^3^/µL	453.8	354.9	0.08	0.079	0.625	0.332

^1^ CS = chute score; ES = exit speed; Cortisol = salivary cortisol concentration; Temperature = rectal temperature; RBC = red blood cell count; HCT = hematocrit; HGB = hemoglobin; WBC = white blood cell count; NEU = neutrophils; LYM = lymphocyte; N:L = neutrophil-to-lymphocyte ratio; MONO = monocytes; EOS = eosinophil; PLT = platelets. ^2^ The values correspond to non-transformed means; however, SEM and *p*-values correspond to GLIMMIX analysis using napierian log transformation. ^3^ Treatments were applied topically (5 mL/site to the nuchal area and muzzle). mBAS = 10 mL maternal appeasing substance (*n* = 11); CT = 10 mL water, used as control (*n* = 11). ^4^ T: treatment; D: day. Main effect and interaction were considered significantly different at *p* < 0.05, while 0.05 < *p* ≤ 0.10 was considered a tendency.

**Table 6:** Net gain outcomes used for the return-on-investment (ROI) calculation for Angus-influenced heifers housed in feedlot pens over a 28-day period receiving (mBAS) or not (CT) a maternal bovine appeasing substance at weaning, immediately prior to a 1 h transportation.; adapted from Pickett et al. [17].

Item ^1^	Net Gain ^2^		Difference ^3^
	mBAS	CT	
Initial BW, kg/pen	3420.46	3446.92	−26.46
Final BW, kg/pen	3943.98	3562.97	381.02
Medication, $/pen	205.54	253.25	−47.71
Feed, $/pen	625.28	667.76	−42.47
Initial value, $/pen	13,134.57	13,236.18	−101.60
Final value, $/pen	15,144.89	13,681.79	1463.11
Profit, $/pen	1179.50	628.03	551.46
ROI, %	1174.07	---	---

^1^ Net gain metrics: Medication = sum of the medication costs within each treatment pen during the entire experimental period. Feed = sum of feed costs within each treatment pen during the entire experimental period. Initial and final BW = sum of the body weight within each treatment pen obtained at weaning (prior- and post-transportation; initial) and at the end of the experimental period (final). Initial and final values = calculated using the summing of pen body weight and adding a 4% shrink, and $4.00/kg for initial and final values. Profit = estimated as final value—(initial value + feed cost + medication cost) [17]. ROI = (profit difference/treatment cost) × 100; considering 11 doses per pen, which amounts to CAD$46.97 per pen (mBAS cost = approximately CAD$4.27 per 10 mL dose × 11 animals per pen). ^2^ Treatments were applied topically (5 mL/site to the nuchal area and muzzle). mBAS = 10 mL maternal appeasing substance (*n* = 11); CT = 10 mL water (*n* = 11). ^3^ Difference in net gain (mBAS in relation to CT).

## Data Availability

The raw data supporting the conclusions of this article will be made available by the authors on request.

## Abbreviations

The following abbreviations are used in this manuscript:

ADG	Average daily gain
ADG_0–14_	Average daily gain calculated between days 0 and 14 post-weaning
ADG_14–27_	Average daily gain calculated between days 14 and 27 post-weaning
ADG_0–27_	Average daily gain calculated between days 0 and 27 post-weaning
BIC	Bayesian Information Criterion
BRD	Bovine Respiratory Disease
BW	Body weight
BW14	Body weight obtained on day 14 post-weaning
CBC	Complete blood cell count
CS	Chute score
CT	Control
DMI	Dry matter intake
DMI_0–13_	Dry matter intake obtained between days 0 and 13 post-weaning
DMI_14–27_	Dry matter intake obtained between days 14 and 27 post-weaning
DMI_0–27_	Dry matter intake obtained between days 0 and 27 post-weaning
EDTA	Ethylenediaminetetraacetic acid
EOS	Eosinophils
ES	Chute exit speed
Final	Measurement obtained on day 27 post-weaning
G:F	Gain-to-feed ratio (feed efficiency)
G:F_0–14_	Gain-to-feed ratio calculated between days 0 and 14 post-weaning
G:F_14–27_	Gain-to-feed ratio calculated between days 14 and 27 post-weaning
G:F_0–27_	Gain-to-feed ratio calculated between days 0 and 27 post-weaning
HCT	Hematocrit
HGB	Hemoglobin
Initial	Measurement obtained on day 0 (weaning)
LYM	Lymphocytes
mBAS	Maternal bovine appeasing substance
MONO	Monocytes
NEU	Neutrophils
N:L	Neutrophil-to-lymphocyte ratio
OCSF	Olds College Smart Farm
PLT	Platelets
RBC	Red blood cell
RFID	Radio frequency identification
ROI	Return-on-investment
TMR	Total mixed ration
U.S.	United States of America
WBC	White blood cell
